# Exploring the Structural and Functional Diversity among FGF Signals: A Comparative Study of Human, Mouse, and Xenopus FGF Ligands in Embryonic Development and Cancer Pathogenesis

**DOI:** 10.3390/ijms24087556

**Published:** 2023-04-20

**Authors:** Ravi Shankar Goutam, Vijay Kumar, Unjoo Lee, Jaebong Kim

**Affiliations:** 1 Department of Biochemistry, Institute of Cell Differentiation and Aging, College of Medicine, Hallym University, Chuncheon 24252, Republic of Korea; 2 iPS Bio, Inc., 3F, 16 Daewangpangyo-ro 712 Beon-gil, Bundang-gu, Seongnam-si 13522, Republic of Korea; 3 Department of Electrical Engineering, Hallym University, Chuncheon 24252, Republic of Korea

**Keywords:** fibroblast growth factors, diversity, diseases, therapeutics, vertebrate models

## Abstract

Fibroblast growth factors (FGFs) encode a large family of growth factor proteins that activate several intracellular signaling pathways to control diverse physiological functions. The human genome encodes 22 FGFs that share a high sequence and structural homology with those of other vertebrates. FGFs orchestrate diverse biological functions by regulating cellular differentiation, proliferation, and migration. Dysregulated FGF signaling may contribute to several pathological conditions, including cancer. Notably, FGFs exhibit wide functional diversity among different vertebrates spatiotemporally. A comparative study of FGF receptor ligands and their diverse roles in vertebrates ranging from embryonic development to pathological conditions may expand our understanding of FGF. Moreover, targeting diverse FGF signals requires knowledge regarding their structural and functional heterogeneity among vertebrates. This study summarizes the current understanding of human FGF signals and correlates them with those in mouse and Xenopus models, thereby facilitating the identification of therapeutic targets for various human disorders.

## 1. Introduction

Fibroblast growth factors (FGFs) were first identified in 1973 in bovine pituitary extract [1]. FGFs represent a family of conserved polypeptide mitogens known for their ability to promote proliferation of various cells [2]. FGFs have pleiotropic activities that distinguish this family from other growth factors. Moreover, combined with their proliferation activity, FGFs manifest neurotrophic and angiogenic activities [3,4] and are involved in developmental events, including differentiation, migration, morphogenesis, and patterning [5]. Abnormal FGF signaling causes various human diseases, such as congenital craniosynostosis, dwarfism syndrome, insulin resistance, obesity, and cancer [6].

FGFs—present in invertebrates and vertebrates—are highly conserved in gene structure and amino acid sequence. Additionally, FGFs are the most diverse group of growth factors in vertebrates; approximately 22 members of FGFs have been identified in vertebrates, with a molecular mass of 17–34 kDa and 13–17% amino acid identity [7]. Most FGF ligands share 28 highly conserved and six identical amino acid residues, implying similarity in the internal core [8]. Based on sequence homology and phylogeny, vertebrate FGFs are subdivided into canonical, hormone-like, and intracellular subfamilies [9]. Members of these subfamilies share high sequence identity and biochemical and developmental properties [10]. Four distinct high affinity receptor tyrosine kinases mediate the effects of FGF signaling [2].

Moreover, diversified FGF signaling requires the precise regulation of FGF activity and receptor specificity. The FGF family is extensively complex [2], and structurally diverse FGF ligands are functionally different [11]. However, a decade-long characterization of the structural and functional diversity within the FGF ligand family has yielded new insights on the differences in the mechanisms of action among members of the FGF family. In this review, we provide an overview of the structural and functional diversity of FGFs in vertebrates, present the supporting evidence for their roles in the pathogenesis of diseases, and discuss potential options for targeting them to develop novel therapeutic approaches.

## 2. Gene Organization and Protein Length

Most *Fgf* genes are dispersed throughout the vertebrate genome. The human and mouse *Fgf* families comprise 22 members, and the Xenopus *Fgf* family codes for approximately 19–20 FGFs [7,12,13]; their chromosomal locations are listed in Table 1. The location of Human *Fgf15*, mouse *Fgf19*, Xenopus *Fgf15*, *Fgf17*, *Fgf18*, and *Fgf21* has not yet been identified. Moreover, evolutionarily, *Fgf15* and *Fgf19* are orthologs in vertebrates; human *Fgf19* and mouse *Fgf15* share 51% amino acid identity, and Xenopus *Fgf19* and mouse *Fgf15* share 59% identity. Additionally, a few *Fgf* genes are clustered in the vertebrate genome, including *Fgf3*, *Fgf4*, and *Fgf19* (*Fgf15* in mice), grouped on chromosome 11 in humans and chromosome 7 in mice. However, these clustered associations of *Fgfs* are common in lower vertebrates, such as Xenopus; *Fgf3*, and *Fgf4*, and *Fgf19* are closely linked on chromosome 4, and *Fgf1*, *Fgf6*, *Fgf7*, and *Fgf23* are grouped on chromosome 3. Notably, *Fgf3*, *Fgf4*, and *Fgf19* are separated by 30 kb and 45 kb on chromosome 4 in Xenopus; however, this distance reduces to 40 kb and 10 kb in human chromosome 11. In humans and Xenopus, these gene locations indicate a conserved evolutionary pattern conferred by gene and chromosomal duplication and gene translocation.

**Figure 1:**
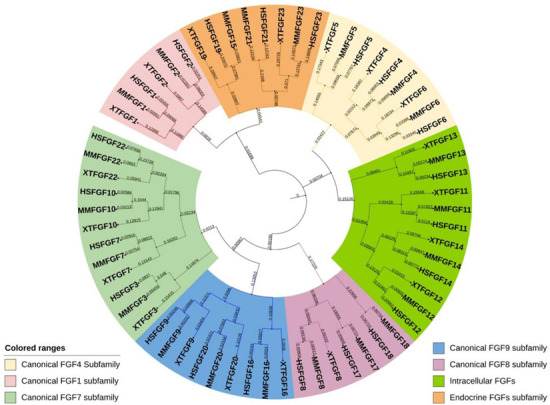
Evolutionary analysis of FGF proteins in humans, mice, and Xenopus. A circular rooted phylogenetic tree, depicting evolutionary relation between FGF proteins in *Homo sapiens* (HS), *Mus musculus* (MM), and *Xenopus tropicalis* (XT) was determined by using Clustal Omega and visualized by iTOL The evolutionary range among each protein is proportionate to the branch lengths and different subfamilies of FGF proteins are indicated by different color ranges.

Prototypic *Fgfs* consist of three coding regions (exons), and this number is relatively conserved in humans, mice, and Xenopus. Exon 1 mainly contains the start codon (ATG); however, there are few *Fgfs* (*Fgf2* and *Fgf3*) where the sequence initiates from an additional 5′-transcribed sequence upstream of ATG [14,15]. Additionally, sub-exons are formed in some *Fgfs* during the splicing process of Exon 1. The gene size of *Fgfs* varies from <2 kb (in *Fgf21*) to over 500 kb (in *Fgf14*). Moreover, unlike other *Fgf* genes, the *Fgf8* exon 1 is subdivided into four small exons in mammals [16] followed by typical exons 2 and 3, reflecting the multifunctionality of the *Fgf8* gene. Comparing the genomic sequence of *Fgf8* genes from various species reveals that the last three exons are substantially conserved despite the upstream exons being very diverse [16]. Based on the phylogeny chromosomal location (synteny) and homology, the *Fgf* gene family in humans, mice, and Xenopus can be categorized into seven subfamilies [17], including *Fgf1*, *Fgf4*, *Fgf7*, *Fgf8*, *Fgf9*, *Fgf11*, and *Fgf19/15* (Figure 1). Phylogenetic studies suggest potential evolutionary and transformative relationships within the vertebrate gene family. Moreover, studying gene loci on chromosomes allows the evaluation of more precise evolutionary relationships within the *Fgf* gene family. Lastly, the protein length of FGF is in the range of 126–268 amino acids (aa) in vertebrates, and FGFs in vertebrates are mostly of similar size; therefore, they are predicted to be similarly structured (Table 2).

## 3. Structural and Functional Diversity

**Figure 2:**
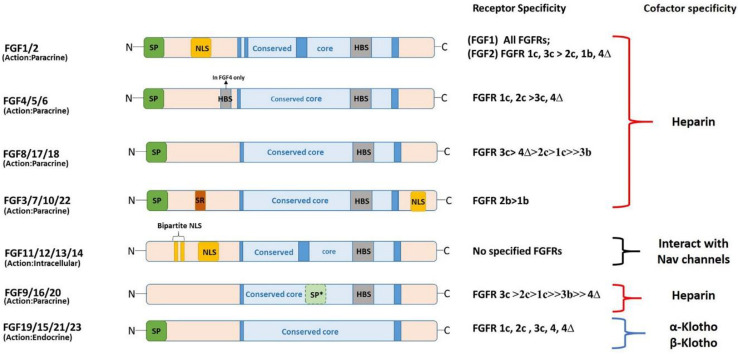
Diagrammatic representation of domain structure of FGF protein. Members of FGF family are classified based on their mode of action. N: amino terminus, SP: signal peptide, NLS: nuclear localization signal, HBS: heparin binding site, SP*: uncleaved bipartite signal sequence, C: carboxyl terminus, SR: serine rich motif.

The molecular weight of FGFs in vertebrates ranges from 17–34 kDa, and the domain structure of FGF protein constitutes an internal core region of approximately 120–140 aa [18] (Figure 2). Within this core region, most FGFs contain a highly conserved sequence of 28 residues and 6 identical amino acids [8]. Among the 28 highly conserved amino acid residues, 10 mediate the interaction of FGFs with their receptors (FGFR) [19]. Moreover, the core region in most FGFs is composed of a cylindrical barrel resulting from the precise folding of 12 antiparallel β-strands. However, FGF1 and FGF2 are exceptions because their structures have a triangular array formed by the typical arrangement of four β-strands [20]. Notably, FGF1 and FGF2—previously known as acidic and basic FGFs—were the first FGFs identified [21]. Sharing 55% homology within their sequence, acidic and basic FGFs have acidic and basic isoelectric points of 5.6 and >9, respectively. FGF1 is a non-glycosylated polypeptide that forms a 17–18 kDa protein (155 aa in length) in most vertebrates [22]. Moreover, the domain structure of vertebrate FGF1 contains a nuclear localization signal (NLS) peptide vital in DNA synthesis. Furthermore, in humans and mice, FGF2 is secreted as a monomer, and it forms multiple isoforms due to the presence of different start codons; however, only one FGF2 variant is known in Xenopus. Additionally, FGF2 functions intracellularly and extracellularly in mammals. The vertebrate FGF3 domain structure is similar to that of other FGFs, except for the presence of a NLS motif at the C-terminal region [23].

Additionally, FGF5 (FGF3a in mice) is a precursor polypeptide containing signal and mature peptides of 17 and 251 aa, respectively, in humans. Unlike other FGFs, FGF5 is characterized by two types of glycosylation: N- and O-linked glycosylation. Lastly, FGF5 has been identified in the *Xenopus tropicalis* genome [24].

However, its chromosomal location is unknown. Alternatively, spliced forms of FGF8 (FGF8a and FGF8b) are highly conserved and well studied in humans, mice, and Xenopus [25,26,27]. The domain structure of FGF9 does not contain any signal peptides. However, FGF10 (keratinocyte growth factor-2) comprises a serine-rich motif positioned at the amino terminus and a long signal peptide [28].

The domain structure of FGF11—known as FGF homologous factor 3 (FHF-3)—maintains an NLS without signal peptides. Additionally, the amino acid terminus of FGF12 (FHF-1) has two NLS sequences known as bipartite NLS (Figure 2). Moreover, FGF14 contains an additional bipartite NLS and signal motif. Since FGF16 and FGF20 lack the usual signal sequence of FGF-9, they are secreted similarly to FGF9. Next, FGF19, FGF21, and FGF23 lack a heparin-binding site within their domain structure. Lastly, FGF18 is a secreted glycosylated polypeptide that interacts with heparin molecules [28] (Figure 2).

Based on the mechanism of action, FGFs can be classified into three subfamilies: canonical (paracrine), endocrine (hormone-like), and intracellular FGFs (Figure 1 and Figure 2). The canonical subfamily has five members of FGFs; however, endocrine and intracellular subfamilies have one member each [10]. The evolutionary relationship indicates that intracellular FGFs may be the first members of the family to evolve, followed by canonical FGFs, and the recent evolutionary trend of endocrine FGFs considered the latest [10].

Furthermore, in the canonical subfamily, FGFs are mainly secreted ligands and are tightly bound to heparin/heparin sulfate (HS) proteoglycans (HSPGs) that regulate their receptor specificity and affinity [29]. Members of this subfamily (FGF1, FGF4, FGF7, FGF8, and FGF9) bind to cell surface FGFRs and their cofactor protein HS to form a FGF: FGFR: HS dimer, activating in vertebrates [30]. Notably, the FGF1 and FGF2 belong to the FGF1 subfamily.

The four major signaling pathways activated by canonical FGFs include the RAS-MAPK, phosphatidylinositol-4,5-bisphosphate 3-kinase-AKT, phospholipase Cγ/protein kinase C, and signal transducer and activator (STAT) pathways [10]. Additionally, canonical FGFs are key regulators of mesenchymal and epithelial signaling required for organogenesis [31].

After binding FGFR, FGF1 crosses the plasma membrane, passes through the cytosol, and reaches the nucleus [32,33]. Notably, FGF1 is the only FGF that can activate all splice variants of FGFR [10], and nuclear FGF1 possibly controls the cell cycle, cell differentiation, survival, and apoptosis [34,35]. Furthermore, Xenopus FGF2 has been identified and cloned, and its spatial and temporal expression suggests its role in early development, especially during neurulation [24]. Additionally, FGF1 and FGF2 are implicated in organogenesis and reportedly promote lens formation and retinal pigment epithelium in Xenopus [36,37]. Lastly, FGF1 and FGF2 in Xenopus activate MAP kinase differently [38].

The FGF4 subfamily comprises FGF4, FGF5, and FGF6 [13]. The presence of FGF5 in this group is controversial due to its close relationship (synteny) with the FGF1 subfamily [1]. Moreover, all members of this family have secreted proteins that possess cleavable N-terminal signal peptides, and they activate IIIc splice variants of FGFRs 1–3 and FGFR4 [39,40].

Phylogenically, the FGF7 subfamily includes FGF3, FGF7, FGF10, and FGF22 [13]. However, some controversies exist regarding the inclusion of FGF3 in this subfamily, as chromosomal synteny supports its inclusion in the FGF4 subfamily [1]. Notably, a recent study has proposed a new subfamily of FGF3 [41]. All members of the FGF7 subfamily selectively activate splice variant IIIb of FGFR2; besides this function, FGF3 and FGF10 activate the IIIb variant of FGFR1 [39,40].

Furthermore, members of the FGF8 subfamily (FGF8, FGF17, and FGF18) contain a cleaved signal peptide at the N-terminus. Additionally, they interact with the IIIc splice variants of FGFRs 1–3 and FGFR4 [39,40].

Alternatively, members of the FGF9 subfamily (FGF9, FGF16, and FGF20) lack any N-terminal signal peptide; however, they comprise an internal sequence that functions as a non-cleaved signal for their movement inside the cytosol and secretion from cells [42,43]. Additionally, this family has the unique property of activating the IIIb variant of FGFR3, FGFR4, and IIIc splice variants of FGFR1, FGFR2, and FGFR3 [39,40].

Endocrine or hormone FGFs (hFGFs)), such as FGF19, have an overall systemic function [44]. Additionally, they have a lower affinity for HS and require protein cofactors αKlotho, βKlotho, or KLPH for binding with their receptors [45]. FGF19/15, FGF21, and FGF23 belong to this group and exert their effects in an FGF-dependent manner. Moreover, endocrine FGFs are involved in bile acid, carbohydrate, lipid, and vitamin D metabolism [9]. FGF21 directly regulates hepatocyte and adipocyte metabolism by interacting with FGFR1 and βKlotho [46,47,48], and FGF19 interacts with and activates FGFR4 and regulates bile acid synthesis and hepatocyte proliferation [46,49]. Additionally, FGF19 is linked to the progression of hepatocellular carcinoma [50], and FGF23 mediates its effect by activating FGFR1c, FGFR3c, FGFR4, and the α-Klotho cofactor [51,52]. Intracellular FGFs (iFGFs), including FGF11, FGF12, FGF13, and FGF14, share a common structural core with other FGFs and have an NLS; however, they are not secreted and do not interact with FGFR [53,54]. They mainly interact with proteins, such as members of the voltage-gated sodium channel family [55], mitogen-activated protein kinase-interacting protein [56], β-tubulin [57] and NF-κB essential modulators [58]. Additionally, FGF13 interacts with microtubules. Other interacting proteins include the MAP kinase scaffolding protein IB2, which interacts with FGF12 (FHF1). Loss of function studies has demonstrated iFGFs involvement in neuronal-related activity [59]. Moreover, studies on chicken, mouse, and Xenopus models have demonstrated that FGF signaling is crucial for mesoderm specification, neural induction, and anterior–posterior axis patterning [60,61,62,63].

## 4. FGF Signaling in Early Development

### 4.1. FGF and Mesoderm Specification

Earlier investigations in the 1990s on Xenopus and other vertebrate models showed that FGF signaling is necessary for the formation of the axial (which later forms the notochord) and paraxial mesoderms (which develops into the axial skeleton, muscles, and dermis) [60,64]. Inhibiting FGF signaling by expressing a dominant negative form of the FGF receptor (Dn-FGFR) disrupts the notochord and somites [60,64,65]. It is unclear whether FGF functions during the induction of axial and paraxial mesoderm or it is required for the maintenance of these mesodermal subtypes. Fletcher and Harland [65] reported this dilemma in 2008, when they showed in their investigation that the induction of the paraxial mesoderm requires FGF, and axial mesoderm only requires FGF for maintenance during gastrulation. The FGF requirement for notochord development is evolutionarily conserved in vertebrates [66]. Additionally, FGF2 (basic FGF) and FGF4 (previously known as eFGF) are mainly implicated in the mesodermal specification of Xenopus embryos [67,68]. Mice and rabbits show similar functions for FGF1 and FGF2 in defining mesodermal specification [69,70]. Additionally, disturbing FGF4 signaling counteracts mesodermal induction in embryonic stem cells [71]. Several independent investigations have demonstrated that FGF signaling is a crucial signaling pathway in vertebrate mesoderm differentiation [60,72]; however, the molecular mechanism by which FGFs regulate mesodermal specification is not entirely understood.

### 4.2. FGF and Neural Specification

The spinal cord cells in vertebrates are derived from neuromesodermal progenitors (NMP) with neural and mesodermal features [73,74]. Events of spinal cord development constitute complex processes, such as neurogenesis, ventral patterning, neural crest specification, and migration, governed by the elongation of the caudal axis [75]. Additionally, spinal cord specification involves the FGF signaling pathway as a key regulator. During chicken spinal cord specification, FGF3, FGF4, FGF8, FGF13, and FGF18 are expressed in the caudal NMP region and tissues surrounding the NMPs [76,77]. FGF8 and FGF4 expression in the NMP region is sustained for several days, and then declines during the last stage of somitogenesis and the cessation of axis elongation [75]. Similar investigations have been performed in mice, where FGF3, FGF4, FGF8, and FGF17 were found in and around the NMP region [78,79,80].

FGF/Ras/Mapk/Ets initiate neural induction in ascidians, which are the last common ancestor of vertebrates in chordate evolution [72,81]. Studies in Xenopus embryos have set the foundation for the classical model (default model) of neural induction, which suggests that signals from the organizer instruct the ectoderm towards neural fate [82]. However multiple investigations in chick embryos have established that FGF signaling is vital in early neural differentiation, challenging the default model idea [83,84]. FGF signaling in neuronal specification can be projected in two ways: first, as an instructive signaling where FGF activates neural genes; second, as antagonist signaling where FGF inhibits BMP signaling via smad1 phosphorylation [12]. Furthermore, Xenopus FGF2 induces the neural-specific gene *Zic3* when expressed ectopically [85]; however, FGF4 of Xenopus was shown to activate early neural markers (zic3, zic1, and foxd5a) and inhibit BMP [86].

Studies indicate that the FGF4-ERK1/2 pathway is crucial for neural specification in embryonic stem cells [87,88] and FGF4 disruption antagonizes neural induction in ES cells [71]. Moreover, midbrain development in chicks [89] and anterior–posterior patterning in Xenopus [26] are significantly influenced by FGF8. Recent findings suggest that FGF2, FGF8, and Ets in Xenopus ectoderm cells are crucial for neural induction both in vivo and in vitro [90]. Hongo et al. [90] showed that neural induction in ectoderm cells was transduced through Fgf/Ras/Mapk/Ets without BMP signal inhibition, consistent with previous studies.

In mice, FGF functions in neural stem cell maintenance and neurogenesis [91]. Additionally, FGF2 and epidermal growth factors can stimulate proliferation and the self-renewal of neural stem cells in vitro [92,93,94,95,96]. FGF2 transforms embryonic stem cells into neural stem cells, defined by self-renewal and the ability to generate neurons, oligodendrocytes, and astrocytes [75]. Moreover, FGF2 in rodents can stimulate functional recovery following spinal cord injury [97,98,99,100] and is involved in reviving synaptic connections [101]. Lastly, FGF22 reportedly regulates excitatory synaptic contact formation [102], and mouse FGF7 is essential for inhibitory synapse formation in the developing hippocampus [103].

### 4.3. FGF Signaling in Metabolism and Diseases (Cancer)

FGF signaling plays a part in the development of almost every organ (including the heart, lungs, brain, urinary system, muscle, skeleton, and skin) and processes such as angiogenesis and lymphangiogenesis [6]. Moreover, endocrine FGFs are functionally essential for metabolism and regulate the brain, kidney, liver, and adipose tissues. The dysregulation of FGF signaling leads to various genetic disorders, including cancer, chronic obstructive pulmonary disease, and chronic kidney disease. The next section briefly reviews the roles of FGFs in metabolism and cancer.

#### 4.3.1. FGF Signaling in Metabolism

FGF15/19, FGF21, and FGF23, which belong to the FGF19 subfamily, are endocrine hormones that regulate bile acid, fatty acid, glucose, and mineral metabolisms. Moreover, FGF19 in humans and its ortholog FGF15 are gut-derived circulating hormones that suppress hepatic bile acid via FGFR4 and the cofactor KLB complex [6]. Additionally, FGF15/19 negatively regulates bile acid synthesis and FGF15 deletion in mice upregulates bile acid synthesis by inducing the expression of the rate-limiting and regulating enzyme cholesterol 7α-hydroxylase (CYP7A1) in the liver [104]. However, FGF15 overexpression restricts bile acid synthesis by downregulating CYP7A1 mRNA levels [104]. Furthermore, FGF19 treatment blocks CYP7A1 expression in human hepatocytes in an autocrine/paracrine manner [105,106].

FGF15/19 suppresses liver fat storage; in one study, FGF19 transgenic mice showed low levels of lipogenic enzymes and liver triglycerides [107]. Moreover, FGF19 blocks lipogenic enzyme expression in rat hepatocytes by inducing STAT3 signaling and suppressing peroxisome proliferator-activated receptor-γ coactivator-1β expression [108]. Additionally, FGF19 induces the expression of proteins associated with fatty acid oxidation [109]. Prolonged treatment with FGF19 in vivo reduces lipid accumulation in the liver and prevents diet-induced steatosis [110]. Moreover, in binding to FGFR4 and KLB, FGF15/19 regulates the energy and glucose metabolism in the brain [111,112]. FGF19 functions in the hypothalamus by activating ERK signaling [113]. Therefore, the FGF15/FGF19 pathway provides great prospects for treating diseases associated with bile acids, such as primary biliary cirrhosis and bile acid diarrhea. Furthermore, a study reported a newly engineered variant of FGF19 that was less effective in activating FGFR4 but still positively affected lipid and glucose metabolism [114]. Lastly, by deactivating the STAT3 pathway, another FGF19 variant, NGM282 (M70), maintains the advantageous effects of BA metabolism and is free of murine mitogenic activity [115]. Phase II clinical studies have been conducted to investigate the effects of M70 in individuals with primary sclerosing cholangitis and diabetes mellitus. These investigations offer a method to develop FGF19 as a potential treatment for associated illnesses and injuries.

FGF21 is a hormone that regulates glucose and lipid homeostasis and insulin sensitivity. FGF21 functions by binding to FGFR1c and its co-receptor protein KLB in the liver, brain, and adipose tissues [116]. FGF21 overexpression in mice resists diet-induced obesity [117], and FGF21 can affect weight loss, reduce plasma glucose and triglyceride levels, and boost insulin sensitivity in obese and diabetic vertebrate models without altering the calorie intake [117,118]. The subcutaneous administration of the FGF21 variant (LY2405319) in DIO mice decreased plasma glucose and body weight at a potency comparable to that of FGF21 [119]. Therefore, FGF21 may be an effective therapeutic agent for the treatment of obesity and fatty liver disease. LY2405319 has undergone phase I clinical testing for lower body weight and fasting insulin, and it is notable for enhancing dyslipidemia in individuals with type 2 diabetes [120].

FGF23 is a regulator of phosphate metabolism and is produced mainly by the osteoblasts and osteocytes of bone tissue [121]. Additionally, FGF23 regulates phosphate and vitamin D homeostasis in skeletal tissues [122], and its mutations lead to low serum phosphorus levels, rickets, bone pain, osteomalacia, and short stature [123]. Moreover, FGF23 overexpression in whole mouse, and mouse liver and osteoblasts, results in a low serum phosphate concentration and rachitic bone [124,125,126]. Furthermore, FGF23 regulates sodium and calcium metabolism [6]. Clinical studies have demonstrated that high serum FGF23 concentration can be used to diagnose kidney disease progression, specifically in the initial stages of diabetic nephropathy [127,128]. Furthermore, injection of a human IgG1 mAB (burosumab), which binds to and inhibits the biological activity of FGF23, restored normal phosphate and vitamin D levels in hypophosphatemia mouse models [129]. The results of burosumab’s phase II clinical studies support its use in X-linked hypophosphatemia. A growing understanding of the physiological regulation and function of FGF23 could contribute to elucidating the pathophysiology of illnesses related to bone and mineral metabolism and kidney-related disorders. Moreover, recent investigations have linked FGF23 to the immune system in chronic kidney disease; FGF23 induces TNF-α expression and macrophages in response to immunological stimuli in mice [130], suggesting its role in inflammatory processes.

Several studies have reported the role of FGFs in the regulation of inflammatory responses. FGF1 can intensify inflammatory responses [131] because it is highly expressed in inflammatory cells and tissues. Additionally, FGF1 stimulates IL-2 synthesis and NF-κB induction in T cells [132] to maintain metabolic homeostasis. Moreover, insulin sensitization has been established in mice receiving FGF1 [131]. In diabetic mice without hypoglycemia, a single injection of mouse recombinant FGF1 resulted in significant dose- and insulin-dependent glucose reduction [133]. Additionally, in diabetic mice, recombinant human FGF1 (rhFGF1) restored blood sugar levels to normal [133]. These observations prompted us to consider the therapeutic potential of FGF1 in mediating insulin sensitivity other than inflammatory reactions.

FGF2 is involved in multiple inflammation-related diseases, such as rheumatoid arthritis (Table 3) and multiple sclerosis [134]. HIV infection positively correlates with FGF2 and CD4^+^ T cells [135]. Additionally, FGF2 is associated with the activation of pro-inflammatory chemokines in endothelial cells (Ecs) and the engagement of monocytes and macrophages during angiogenesis [136]. However, few studies have reported the role of FGF3 in inflammation. FGF3 expression significantly upregulated in acute rhinitis and chronic sinonasal inflammation (Table 3) in murine models [137,138]. Overall, associations between canonical FGFs in HIV and pro-inflammatory chemokine regulation may provide an insight into inflammatory disorders, HIV pathogenesis, and responses to their therapy.

#### 4.3.2. FGF Signaling in Various Types of Cancer

FGFs are associated with the initiation and progression of cancers, such as multiple myeloma, urothelial carcinoma, hepatocellular carcinoma, and prostate cancer. The FGF1 expression level in several cancer types, such as breast cancer, hepatocellular carcinoma, and esophageal cancer, shows that growth factors promote tumor cell invasion and metastasis [139,140,141]. A recent study showed that FGF1 regulates colorectal cancer progression (Table 3) through the mTOR-S6K1 dependent pathway [142]. FGF1 association with various cancer types indicates its potential diagnostic and therapeutic importance.

FGF2 can promote the development of breast cancer cells through ligand-independent activation and the recruitment of estrogen receptor α and PRB4δ4 isoform to MYC regulatory regions [143]. Additionally, lung cancer cells that depend on the FGF2/FGFR pathway may be prevented from proliferating using the FGF2 aptamer, which inhibits FGF2 activity [144]. In human melanoma produced as a subcutaneous tumor model in nude mice, introducing an episomal vector encoding antisense FGF2 or FGFR1 cDNA could entirely prevent the formation of tumors by blocking angiogenesis [145]. Targeting FGF2 to limit melanoma angiogenesis results in decisive anti-melanoma effects, which could lead to novel therapeutic approaches for patients with advanced stages of the disease.

FGF4 is expressed more frequently in germ cell cancers, particularly non-seminomas, and may target all-trans-retinoic acid to encourage the growth of malignant-cultured embryonal carcinomas [146]. Moreover, increased FGF4 expression is linked to ovarian cancer (Table 3) stem-like cells’ or cancer-initiating cells’ increased capacity to initiate tumors [147]. Furthermore, FGF5 is highly expressed in patients with breast cancer [148], and FGF6 expression is significantly induced in metastatic liver carcinoma tissues and reduced in non-metastatic liver cancer lesion tissues [149]. Moreover, FGF7 levels are elevated in gastric adenocarcinoma and gastric inflammation [150]. In prostate cancer, FGF8 overexpression is associated with low patient survival [151]. Additionally, as a downstream cell growth regulator, FGF8 can mediate the tumor inhibitory effect of Annexin-A7 in prostate cancer [152]. Moreover, prostate cancer cell proliferation may be significantly reduced by neutralizing antibodies targeting FGF8b [151]. Likewise, the inhibition of FGF5, FGF7, and FGF4 by themselves or in combination with known FGF antagonists may serve as a broad-spectrum therapy for patients with melanoma. Furthermore, FGF9 expression has been observed in many non-small-cell lung carcinoma (NSCLC) primary tumors, and high expression of FGF9 is linked to the low survival rate of patients with NSCLC [153]. Lastly, abnormal FGF10 regulation through FGFR2b and FGFR1b facilitates the progression of prostate cancer, breast cancer, pancreatic adenocarcinoma, gastric carcinoma, skin cancer, and lung squamous cell carcinoma [154]. These findings could provide novel approaches to target FGF9 and FGF10 signaling in various cancers.

Recently, FGF11, as part of a six-gene signature, has been linked to a worse prognosis in bladder cancer [155], and macrophage-specific FGF12 accelerates the development of liver fibrosis in mice [156]. In the future, liver fibrosis and bladder cancer may be treated with therapeutic methods that block macrophage FGF12 and FGF11 expression. Furthermore, FGF13 is highly upregulated in pancreatic endocrine and metastatic breast tumors [157], and FGF13 may enable cancer cells to avoid proteostasis stress induced by oncogene activation.

Compared with normal tissue, primary colorectal cancer has reduced FGF14 expression, and significantly higher methylation of FGF14 has been observed in colorectal cancer [158]. Additionally, FGF14 overexpression dramatically decreased tumor growth in a xenograft mouse model [158]. Therefore, FGF14 is a novel tumor suppressor that functions by regulating the PI3K/AKT/mTOR pathway to inhibit cell growth and induce apoptosis. Furthermore, FGF16 is speculated to contribute to the development of certain cancers including embryonic carcinoma, ovarian cancer, and liver cancer. FGF16 is overexpressed in resected lung cancer tissues, and its high level is inversely correlated with low levels of miR-520b—an inhibitor of cellular migration and invasion [159]. Overall, miR-520b and FGF16 may be helpful in clinical treatment, with FGF16 as a potential biomarker.

In the CD44^+^ subpopulation of colon adenoma cells, FGF18/FGFR3IIIc was elevated, promoting tumor cell proliferation [160]. Additionally, FGF18 downregulation inhibits gastric cancer development, causes G1-phase cell cycle arrest, and improves anticancer treatment sensitivity [161]. These investigations identified FGF18 as a novel prognostic indicator of colon cancer development and a therapeutic target in gastric cancer. Furthermore, FGF10/FGF17 has been identified as a prognostic and drug response marker in acute myeloid leukemia [162], suggesting that small-molecule inhibitors of FGF10 and FGF17 are promising therapeutic targets.

A subset of human hepatocellular carcinomas is driven by abnormal signaling through FGF19 and its receptor FGFR4, which is associated with poor prognosis [163]. Additionally, in humans and mice, FGF19 significantly increases tumor invasiveness caused by the Pregnane X receptor [164]. FGF19 inactivation may be an effective therapeutic strategy for cancers and other malignancies involving the interaction between FGF19 and FGFR4. Moreover, an antibody blocking the interaction of FGF19 to FGFR4 limited the formation of colon tumor xenografts in vivo, preventing hepatocellular carcinomas in FGF19 transgenic mice [165]. For the treatment of liver and colon cancer, and cancers related to head and neck squamous cells, inactivating FGF19 may counteract carcinomas.

Similarly, FGF20 has also been implicated in cancer and is associated with the suppression of macrophage function via β-catenin activation in glioma cells (Table 3) [166]. Furthermore, FGF21 is vital in preventing the onset of advanced diseases, such as pancreatic ductal adenocarcinoma or hepatocellular carcinoma (Table 3), by delaying the onset of the fatty pancreas, steatopancreatitis, fatty liver, and steatohepatitis [167]. Additionally, FGF22 aids pancreatic cancer cell invasion and migration [168]. Hence, developing analogs of FGF21 and antagonists of FGF22 could be therapeutically beneficial for treating chronic liver and pancreas diseases.

FGF23 advances prostate cancer as an autocrine, paracrine, or endocrine growth factor. In vitro studies showed that FGF23 promotes prostate cancer cell line proliferation, invasion, and anchorage-independent growth; however, FGF23 knockdown slows tumor growth in vivo [169]. These investigations on FGF23 demonstrate its multifaceted role in disease progression and how its blockade can be beneficial in eliminating metabolic/mineral, kidney disorders, and cancer regression. Overall, FGF signaling networks are becoming a more appealing target for cancer therapeutic intervention as a result of these studies.

**Table 3:** FGFs and associated diseases, particularly those associated with tumorigenesis.

FGFs	Associated Diseases	References
FGF1	Colorectal cancers, breast carcinoma, hepatocellular carcinoma, and esophagus cancer	[139,140,141,142]
FGF2	Rheumatoid arthritis, multiple sclerosis, breast cancer, lung cancer, and glioblastoma	[134,162]
FGF3	Acute rhinitis and chronic sinonasal inflammation	[137,138]
FGF4	Germ cell carcinoma and ovarian cancer	[146,147]
FGF5	Breast cancer	[148]
FGF6	Liver cancer	[149]
FGF7	Gastric adenocarcinoma and gastric inflammation	[150]
FGF8	Prostate cancer	[151,152]
FGF9	Non-small cell lung carcinoma (NSCLC)	[153]
FGF10	Prostate cancer, breast cancer, pancreatic adenocarcinoma, gastric carcinoma, skin cancer and lung squamous cell carcinoma, and acute myeloid leukemia	[154,162]
FGF11	Bladder cancer	[155]
FGF12	Liver fibrosis	[156]
FGF13	Pancreatic cancer, endocrine cancer, and breast cancer	[157]
FGF15/19	Hepatocellular carcinoma	[170]
FGF16	Embryonic carcinoma, ovarian cancer, and liver cancer	[159]
FGF17	Acute myeloid leukemia	[162]
FGF18	Breast cancer	[171]
FGF20	Glioma	[166]
FGF21	Prevents pancreatic ductal adenocarcinoma or hepatocellular carcinoma	[167]
FGF14	Tumor suppressor in colorectal cancer	[158]
FGF22	Pancreatic cancer	[168]
FGF23	Tumor induced ostomalacia	[172]

## 5. Conclusions

Current understanding of the roles of FGF signaling in various biological and developmental processes has substantially improved in the last few decades. The FGF/FGFR system influences the pathophysiology of numerous human ailments, including hereditary disorders, metabolic diseases, and cancers. Moreover, the molecular structures of FGFs and their specific receptors regulate the transduction specificity and activation of FGF signaling. Therefore, knowledge regarding the structural and functional diversity of FGFs among different species is pertinent to understanding their influence on health and disease progression. In this review, we outlined the structures and functions of several vertebrate FGFs and correlated them with various human disorders.

The precise roles of specific FGFs/FGFRs in the onset and progression of diseases, their spatiotemporal expression patterns, and underlying mechanisms remain largely unclear. During various developmental and pathological processes, an extensive crosstalk occurs between the FGF pathway and other signaling pathways, including the BMP/TGF-β, PTH, hedgehog, and retinoid pathways. Therefore, an understanding of the interaction mechanism of FGF signaling with multiple signaling pathways in different species will provide a molecular foundation for designing combination therapies [173]. Additionally, FGF synthesis and expression can differ among various species. For instance, different species may have specific transcription factors that regulate particular FGF synthesis. Therefore, when analyzing these findings in different models and applying them to human health, it is crucial to consider any potential inter-species variations in FGF biology.

Furthermore, precision medicine considerably relies on biomarkers and genetic variants. Therefore, identifying specific mutations and biomarkers associated with FGF-related diseases will facilitate the development of more targeted treatments. However, the commonly employed technique for determining the contribution of certain FGFs to disease etiology has drawbacks.

We need new methodologies to gain insights into FGF-targeted therapy, including more spatiotemporally programmable genetic methods, single-cell analysis, in vivo imaging, additional species of model organisms, and omics technologies. From the patient care perspective, big data and artificial intelligence (AI) can be used to analyze patient data to find trends that predict the evolution of FGF-related diseases and how well they will respond to therapy.

On the bright side, scientists are approaching these targeted therapies in diverse ways. For example, clinical trials have evaluated several FGF aptamers targeting bone-forming sites in skeletal tissues and small molecule disrupters targeting several cancers. Disrupter drugs such as PD173074 and bemarituzumab are the potent and selective blockers of FGFR1 and FGFR3, and FGFR2b, respectively [174,175], and are used to treat different cancers.

Conclusively, detailed studies on the structure- and function-based drug designing of agonists and antagonists are warranted to improve therapeutic development.

## Figures and Tables

**Table 1:** Chromosomal location of FGF ligands in different vertebrate species. Information on the genome for human, mouse, and Xenopus models has been sourced from NCBI GeneBank, Mouse genome informatics, and Xenbase database, respectively.

*Fgfs*	Human	*Mus musculus*	*Xenopus tropicalis*
*Fgf1*	Chr5: 142,001,623–142,022,227 (20,605 bp)	Chr18: 38,971,725–39,062,532 (90,807 bp)	Chr3: 37,101,483–37,152,750 (51.27 kb)
*Fgf2*	Chr4: 124,206,684–124,278,197 (71,514 bp)	Chr3: 37,402,616–37,464,255 (61,639 bp)	Chr1: 65,933,388–65,960,806 (27.42 kb)
*Fgf3*	Chr11: 69,397,666–69,406,878 (9213 bp)	Chr7: 144,392,349–144,397,085 (4736 bp)	Chr4: 12,654,687–12,775,488 (120.8 kb)
*Fgf4*	Chr11: 69,360,727–69,363,101 (2375 bp)	Chr7: 144,415,123–144,418,982 (3859 bp)	Chr4: 12,805,564–12,818,136 (12.57 kb)
*Fgf5*	Chr4: 81,646,219–81,666,886 (20,668 bp)	Chr5: 98,402,043–98,424,892 (22,849 bp)	Chr1: 95,522,903–95,569,520 (46.62 kb)
*Fgf6*	Chr12: 4,413,569–4,425,041 (11,473 bp)	Chr6: 126,992,505–127,001,681 (9176 bp)	Chr3: 11,179,405–11,197,664 (18.26 kb)
*Fgf7*	Chr15: 47,431,515–47,495,579 (64,065 bp)	Chr2: 125,876,578–125,933,105 (56,527 bp)	Chr3: 104,281,252–104,316,082 (34.83 kb)
*Fgf8*	Chr10: 103,194,668–103,200,244 (5577 bp)	Chr19: 45,724,930–45,742,941 (18,011 bp)	Chr7: 31,012,191–31,022,187 (10 kb)
*Fgf9*	Chr13: 20,043,875–20,074,184 (30,310 bp)	Chr14: 58,308,131–58,350,311 (42,180 bp)	Chr2: 157,463,721–157,503,485 (39.77 kb)
*Fgf10*	Chr5: 44,350,598–44,434,285 (83,688 bp)	Chr13: 118,851,235–118,929,109 (77,874 bp)	Chr1: 194,526,912–194,599,107 (72.2 kb)
*Fgf11*	Chr17: 7,543,254–7,548,814 (5561 bp)	Chr11: 69,686,894–69,693,775 (6881 bp)	Scaffold_2560: 505–2966
*Fgf12*	Chr3: 193,182,711–193,446,925 (264,215 bp)	Chr16: 27,976,535–28,571,995 (595,460 bp)	Chr5: 105,036,268–105,258,133 (221.87 kb)
*Fgf13*	ChrX: 136,419,343–136,499,434 (80,092 bp)	ChrX: 58,107,499–58,630,932 (523,433 bp)	Chr8: 69,689,404–69,876,346 (186.94 kb)
*Fgf14*	Chr13: 100,073,036–100,752,125 (679,090 bp)	Chr14: 124,211,853–124,915,098 (703,245 bp)	Chr2: 119,766,570–120,110,357 (343.79 kb)
*Fgf15*	Missing	Chr7: 1,444,502,269–1,444,454,690 (47,579 bp)	Missing
*Fgf16*	ChrX: 77,447,389–77,457,278 (9889 bp)	ChrX: 104,808,083–104,820,138 (12,055 bp)	Chr8: 45,583,466–45,609,547 (26.08 kb)
*Fgf17*	Chr8: 21,922,365–21,928,256 (5892 bp)	Chr 17: 70,873,643–70,880,064 (6421 bp)	Missing
*Fgf18*	Chr5: 170,827,589–170,865,098 (37,510 bp)	Chr 11: 33,066,970–33,097,400 (30,430 bp)	Missing
*Fgf19*	Chr11: 69,285,937–69,292,036 (6100 bp)	Missing	Chr4: 12,861,360–12,867,681 (6.32 kb)
*Fgf20*	Chr8: 16,860,698–16,870,038 (9341 bp)	Chr 8: 40,732,207–40,739,994 (7787 bp)	Chr1: 42,116,142–42,120,691 (4.55 kb)
*Fgf21*	Chr19: 53,951,306–53,953,289 (1984 bp)	Chr 7: 45,263,314–45,264,914 (1600 bp)	Missing
*Fgf22*	Chr19: 590,926–594,604 (3679 bp)	Chr 10: 79,590,887–79,593,629 (2742 bp)	Chr1: 105,764,076–105,794,176 (30.1 kb)
*Fgf23*	Chr12: 4,347,654–4,359,141 (11,488 bp)	Chr6: 127,049,865–127,059,259 (9394 bp)	Chr3: 11,271,103–11,276,872 (5.77 kb)

**Table 2:** Protein length of FGF ligands in different vertebrate species. Information on each protein for human, mouse, and Xenopus models has been sourced from NCBI GeneBank, Uniprot, and Xenbase databases, respectively.

FGFs	Human (aa)	*Mus musculus* (aa)	*Xenopus tropicalis* (aa)
FGF1	155	155	155
FGF2	155	154	154
FGF3	239	245	236
FGF4	206	202	192
FGF5	268	264	251
FGF6	208	208	195
FGF7	194	194	194
FGF8	233	244	211
FGF9	208	208	208
FGF10	208	209	196
FGF11	225	225	133
FGF12	243	243	243
FGF13	245	245	255
FGF14	247	247	252
FGF15	NA	218	NA
FGF16	207	207	202
FGF17	216	216	NA
FGF18	207	207	NA
FGF19	216	NA	215
FGF20	211	211	208
FGF21	209	210	NA
FGF22	170	162	175
FGF23	251	251	254

## Data Availability

Not applicable.
